# Validation of a Deep Learning-Based Markerless Video System for Gait Speed Assessment During the 10-Meter Walk Test in Outdoor Clinical Settings

**DOI:** 10.3390/jfmk11030357

**Published:** 2026-09-07

**Authors:** Teerawat Kamnardsiri, Phasit Charoenkwan, Sirinun Boripuntakul

**Affiliations:** 1 Department of Digital Game, College of Arts, Media and Technology, Chiang Mai University, Chiang Mai 50200, Thailand; teerawat.k@cmu.ac.th; 2 Department of Modern Management and Information Technology, College of Arts, Media and Technology, Chiang Mai University, Chiang Mai 50200, Thailand; phasit.c@cmu.ac.th; 3 Integrated Neuro-Musculoskeletal, Chronic Disease, and Aging Research Engagement Center (ICARE Center), Department of Physical Therapy, Faculty of Associated Medical Sciences, Chiang Mai University, Chiang Mai 50200, Thailand

**Keywords:** gait speed, 10-meter walk test, artificial intelligence, deep learning, markerless video system, concurrent validity, innovation system, information technology

## Abstract

**Background/Objectives**: Markerless video-based gait assessment has emerged as a promising alternative to laboratory motion capture systems because it enables objective, low-cost, and accessible mobility assessment. However, evidence supporting the validity of deep learning (DL)-based markerless video systems for gait speed assessment during the 10-meter walk test (10-MWT) in real-world clinical environments remains limited. This study evaluated the concurrent validity of a DL-based markerless video system for estimating gait speed during the 10-MWT under outdoor clinical conditions. **Methods**: Thirty-two healthy participants from three age groups completed the 10-MWT under comfortable, slow, and fast walking conditions. Gait speed was simultaneously measured using the proposed DL-based markerless video system with the YOLOv11x model and Tracker video analysis software (version 6.3.2). Gait speed was calculated across six consecutive 1 m walking segments. Concurrent validity was evaluated using Pearson’s correlation coefficients and Bland-Altman analysis. **Results**: Pearson’s correlation coefficients ranged from 0.92 to 0.98 across all six walking segments and walking-speed conditions, indicating very high concurrent validity. Bland-Altman analysis demonstrated good agreement between the proposed system and the reference method, with mean differences close to zero and most observations falling within the 95% limits of agreement. The proposed system maintained high agreement across comfortable, slow, and fast walking conditions while enabling segment-specific gait speed analysis throughout the 10-MWT. **Conclusions**: The proposed DL-based markerless video system demonstrated high agreement with Tracker for gait speed assessment during the 10-MWT under outdoor non-laboratory conditions, supporting its potential as an accessible approach for gait speed assessment.

## 1. Introduction

Gait speed is widely recognized as one of the most robust indicators of functional mobility and overall health in older adults. It has consistently been associated with a broad range of clinically important outcomes, including falls, frailty, cognitive decline, disability, hospitalization, and mortality, making it an essential measure in geriatric assessment and rehabilitation [1,2,3,4,5]. Because of its strong predictive value, gait speed is routinely used to evaluate mobility, monitor disease progression, assess treatment effectiveness, and identify older adults at risk of functional decline.

Among the available clinical assessments, the 10-meter walk test (10-MWT) is one of the most frequently used methods for measuring gait speed because it is simple, inexpensive, highly reliable, and applicable across a wide range of clinical populations, including individuals with stroke, spinal cord injury, Parkinson’s disease, and healthy older adults [6,7,8,9,10]. However, the conventional 10-MWT yields only a single measure of average gait speed, which summarizes walking performance over the entire testing distance. Although this parameter is clinically valuable, it does not describe how walking speed changes throughout the test. Consequently, two individuals may achieve the same average gait speed while exhibiting different temporal patterns of walking speed during the 10-MWT. These temporal characteristics cannot be distinguished when gait performance is represented by a single average value. Therefore, evaluating instantaneous gait speed throughout walking may provide a more comprehensive characterization of walking performance by revealing temporal changes in gait speed beyond those captured by average gait speed alone [11,12].

Laboratory-based motion analysis systems and high-speed video tracking systems can accurately quantify instantaneous gait speed and are widely regarded as the reference standard for gait analysis because of their high measurement accuracy and temporal resolution. However, their widespread clinical implementation remains limited by high equipment costs, specialized laboratory requirements, complex calibration procedures, and time-consuming post-processing. These limitations have driven increasing interest in markerless video-based systems, which combine conventional video cameras with computer vision techniques to estimate human movement. Compared with laboratory-based motion capture systems, these systems are inexpensive, portable, require minimal setup, and are suitable for use in hospitals, outpatient clinics, and community settings. Previous studies have demonstrated excellent validity and test-retest reliability of markerless video-based gait analysis systems for gait speed assessment under controlled laboratory conditions [13,14]. However, the accuracy of these systems may be compromised in real-world environments because conventional computer vision algorithms are highly sensitive to variations in illumination, background complexity, camera position, and environmental conditions, resulting in reduced measurement accuracy outside laboratory settings [15].

Recent advances in deep learning (DL) have substantially enhanced the capability of computer vision systems for markerless human pose estimation. Unlike conventional image-processing approaches that rely on manually designed features, DL-based algorithms automatically learn hierarchical image representations and have demonstrated greater robustness to variations in lighting, background, clothing, body orientation, and camera viewpoints [16,17]. Consequently, DL-based markerless motion analysis has shown promising validity for estimating spatiotemporal gait parameters and lower-limb kinematics relative to laboratory-based motion capture systems, suggesting considerable potential for clinical gait assessment using only a single camera [18,19]. Despite these technological advances, most validation studies have been conducted under highly controlled laboratory conditions or have primarily focused on general gait parameters rather than gait speed assessment during standardized clinical walking tests. To date, evidence regarding the validity of DL-based markerless video systems for quantifying gait speed during the standard 10-MWT in outdoor non-laboratory environments remains scarce. Establishing the validity of such systems is a critical step toward enabling accessible, low-cost, and objective gait assessment for routine clinical practice and community-based rehabilitation.

Therefore, this study aimed to determine the validity of a DL-based markerless video system for gait speed assessment during the 10-MWT performed under outdoor non- laboratory conditions by comparing its measurements with those obtained from a reference video tracking system. We hypothesized that the proposed system would demonstrate excellent agreement with the reference method while providing a practical, low-cost, and accessible solution for objective gait speed assessment outside specialized motion analysis laboratories.

## 2. Materials and Methods

### 2.1. Participants

Thirty-two participants were recruited, including twelve young adults (25–44 years), ten middle-aged adults (45–59 years), and ten older adults (≥60 years). Participants from different age groups were included to evaluate the proposed system across a broad range of walking characteristics. Participants were eligible if they were able to walk independently and safely for at least 10 m without the use of an assistive device and were able to understand verbal instructions and provide written informed consent. Participants were excluded if they had neurological disorders known to affect gait (e.g., Parkinson’s disease, stroke, or multiple sclerosis), acute or chronic musculoskeletal disorders that could impair walking, lower-limb injury or surgery within the previous 6 months resulting in abnormal gait or weight-bearing, vestibular disorders affecting balance, uncorrected sensory impairments (e.g., vision or hearing deficits), or were taking medications known to influence balance (e.g., sedatives or antidepressants).

The study protocol was approved by the Human Ethical Review Board of the primary investigator’s institution (approval number: AMSEC-67EX-096). All participants provided written informed consent before participation.

### 2.2. DL-Based 10-Meter Walk System Setup

**Figure 1:**
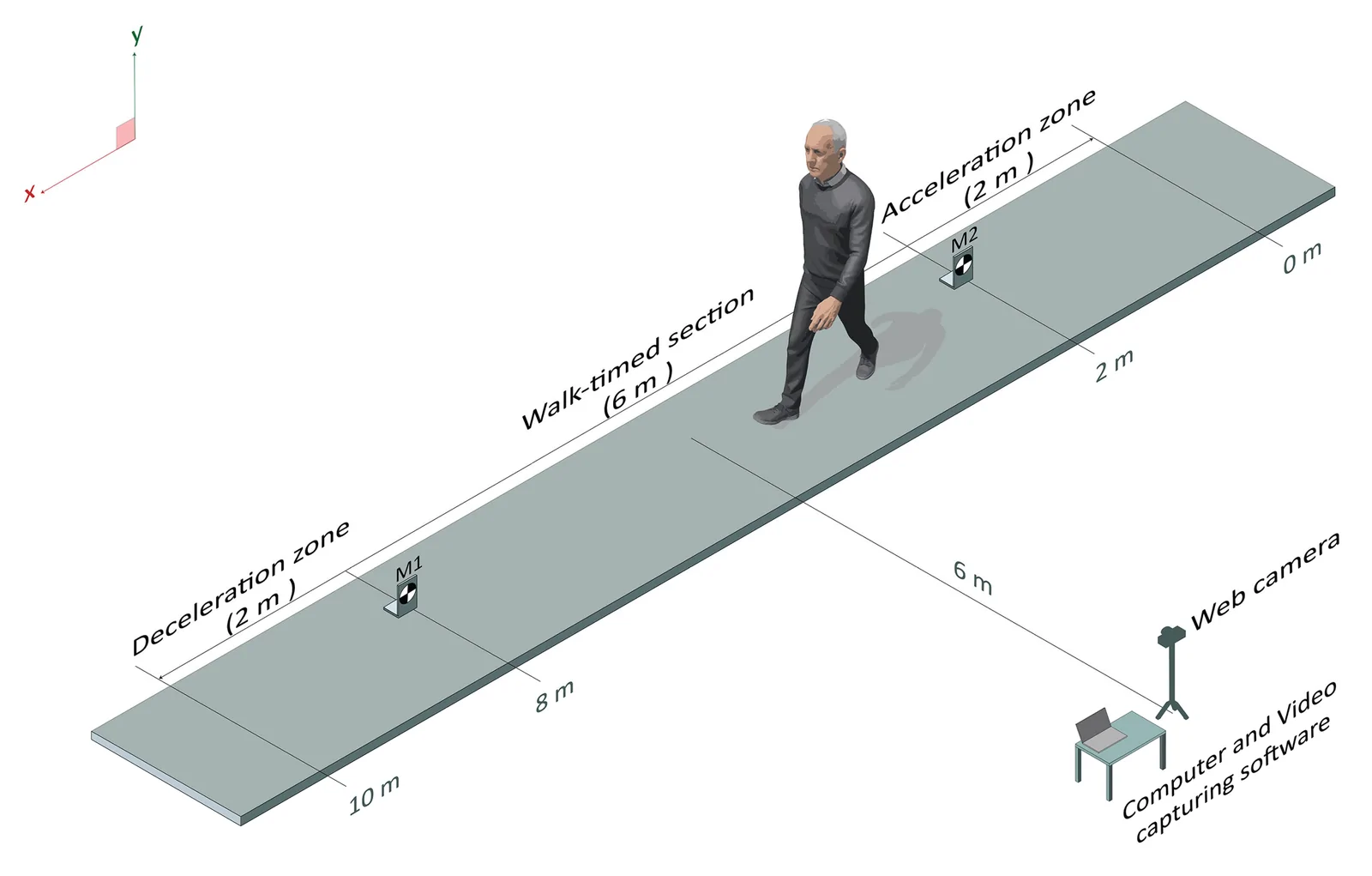
Experimental setup of the DL-based markerless video system for the 10-MWT.

The DL-based markerless video system consisted of a single full high-definition (HD) webcam (Logitech C920, Logitech, Ho Chi Minh City, Vietnam) positioned perpendicular to the participant’s direction of travel at the midpoint of the 10-meter walkway. The camera was mounted at a height of 1.0 m above the ground and positioned 6.0 m from the walkway to ensure full-body visualization in the sagittal plane while covering the entire 10-meter walking path. Video was recorded at a frame rate of 30 Hz with a resolution of 1280 × 720 pixels in MOV format. Two reference markers were placed at the 2 m and 8 m points of the walkway, defining the central 6 m timed section used for gait speed measurement. An external synchronization box was positioned beside the participant’s chair to synchronize the recordings of the proposed system with those of the reference video tracking system. The experimental setup is illustrated in Figure 1.

### 2.3. Assessment of the Concurrent Validity of the DL-Based Approach Gait Speed Detection

Baseline demographic characteristics, including age, gender, height, weight, medical history, and history of falls during the previous 12 months, were recorded before testing. Reflective markers (5 cm in diameter) were attached to the left and right lateral aspects of the trunk to ensure continuous tracking by the reference video tracking system during both forward and return walking trials. All assessments were conducted on level outdoor walkways in non-laboratory assessment environments to evaluate the proposed system under conditions representative of routine gait assessment outside laboratory settings. Data collection was conducted during daytime hours across multiple outdoor non- laboratory locations, including an outdoor activity area in front of a building, a walkway adjacent to a building, a parking area, and a community multipurpose pavilion. Natural lighting conditions varied across testing sessions, ranging from bright sunlight to less intense daylight, with corresponding variations in shadows and surrounding backgrounds. The camera was mounted on a tripod throughout data collection to maintain stability and standardized positioning. Before testing, participants completed one familiarization trial at each walking speed condition (comfortable, fast, and slow) to become accustomed to the testing procedures.

Participants performed the 10-MWT according to the standardized protocol [20,21]. Each trial began with the participant standing at the starting line. Upon the verbal command of “Go”, participants walked continuously along the 10-meter walkway and stopped after crossing the finish line. The central 6 m section, defined by markers placed at the 2 m and 8 m points of the walkway, was used for gait speed measurement to minimize the effects of acceleration and deceleration. For the comfortable-speed condition, participants were instructed to walk at their usual everyday walking speed, whereas for the fast-speed condition, they were instructed to walk as fast as possible without running. To evaluate the proposed system across a broader range of gait speeds, an additional slow-speed walking condition was incorporated into the testing protocol. During this condition, participants were instructed to walk as slowly as possible while maintaining a natural and comfortable gait. Each participant completed two trials under each walking speed condition, resulting in a total of six walking trials. The order of the three walking speed conditions was randomized to minimize potential order effects. A 1 min rest period, or longer if required, was provided between consecutive trials to minimize fatigue. Throughout all walking trials, gait speed was recorded simultaneously using the proposed DL-based markerless video system and the reference video tracking system.

### 2.4. DL-Based Gait Speed Detection for the 10-MWT

**Figure 2:**
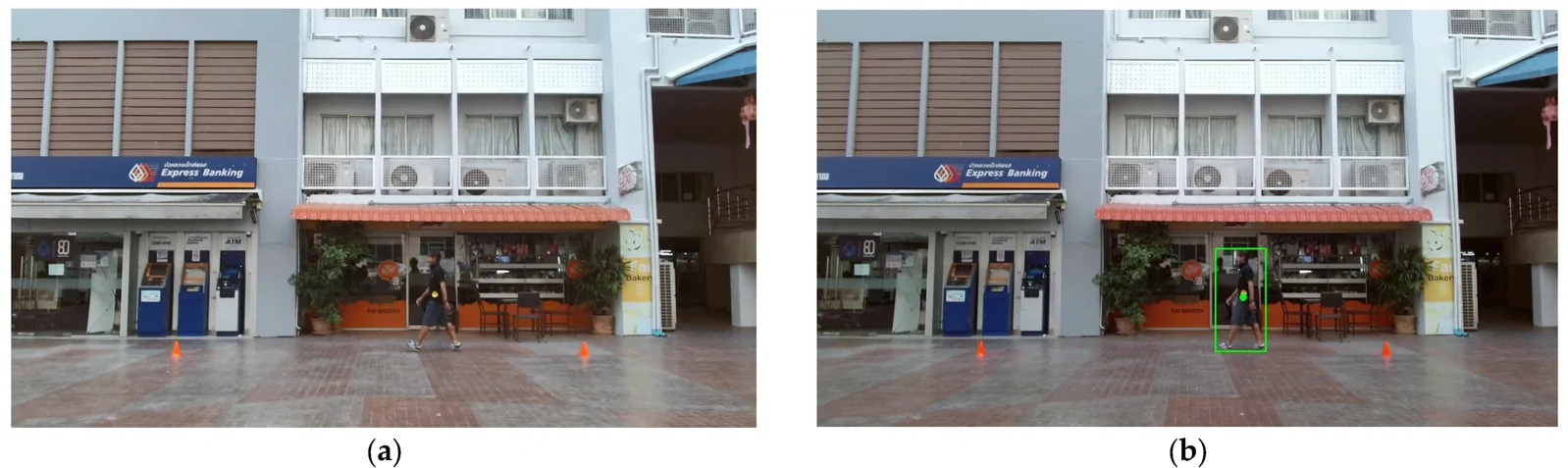
Example video frames used in the proposed DL-based gait speed detection system. (**a**) Original video frame captured during the 10-meter walk test. (**b**) Participant detected by the proposed YOLOv11x object detection model.

The proposed DL-based markerless video system estimated gait speed during the 10-MWT through a five-step processing pipeline: (1) capture volume calibration, (2) human body detection, (3) body position estimation, (4) instantaneous gait speed calculation, and (5) gait speed segmentation. Figure 2 illustrates an example of the captured video frame and the corresponding human body detection result generated by the proposed system.

Step 1: Capture volume calibration

The first step involved calibration of the capture volume using two reference markers (*M*_1_ and *M*_2_) positioned at the beginning and the end of the 6 m measurement zone (Figure 1). These markers, located 6.0 m apart, were used to determine the horizontal calibration distance (x-axis) and establish the pixel-to-distance conversion required for subsequent gait speed estimation. This calibration provided a one-dimensional (horizontal) pixel-to-distance conversion, which was considered appropriate given the fixed, tripod-mounted camera positioned perpendicular to a standardized, straight-line walkway. Lens distortion correction, homography-based perspective correction, and full camera calibration (intrinsic and extrinsic parameters) were not performed in this study; this methodological simplification is addressed as a limitation in Section 4.

Step 2: Human body detection.

The second step involved detection of the participant using the pretrained YOLOv11x object detection model (Ultralytics). The model, pretrained on the MS COCO dataset, was employed directly in inference mode without additional fine-tuning or architectural modification. The default inference settings provided by Ultralytics were used without study-specific modification. No video recordings from participants in the present study were used for model training; therefore, no study-specific training, validation, or test split was performed. For each video frame, the model identified the participant and determined the centroid of the detected body region. The centroid was subsequently used as the reference point for estimating the participant’s horizontal position and tracking body movement throughout the walking trial. Figure 2 presents an example of the original video frame and the corresponding participant detection generated by the YOLOv11x model. The bounding-box centroid, rather than the anatomical center of mass, was used as the position reference. This centroid-based approach was adopted because, during straight-line over-ground walking, forward body translation is predominantly reflected in the horizontal displacement of the whole-body bounding box, and because gait speed was derived from displacement across multiple frames rather than from instantaneous position alone, which is expected to attenuate the influence of local frame-to-frame fluctuations related to arm swing, trunk motion, clothing, or partial occlusion. This simplification relative to joint-center or marker-based tracking is acknowledged as a limitation in Section 4.

Step 3: Body position estimation

The third step involved estimation of the participant’s horizontal position while walking. The calibration parameters obtained from *M*_1_ and *M*_2_ were used to convert the centroid coordinates from image pixels into real-world coordinates. Specifically, the centroid position along the horizontal axis (*ActualPos_X*) was calculated using Equations (1)–(4).

(1) $${CalVolume}_{X}=M_{2}-M_{1}$$

(2) $${OnePixel\_X}_{Distance}=\frac{6}{CalVolume\_X}$$

where 6 is the actual distance between *M*_1_ and *M*_2_ (6.0 m)

(3) $${Body\_X}_{Pixel}=x_{centroid}-M_{1}$$

(4) $$ActualPos\_X={Body\_X}_{pixel}\times {OnePixel\_X}_{Distance}$$

Step 4: Instantaneous gait speed calculation

The fourth step involved the calculation of instantaneous gait speed. The centroid positions obtained from each video frame were stored sequentially as a time-series array. Instantaneous gait speed along the horizontal axis (*Speed_inx_*) was calculated every four consecutive video frames (*DurationTime =* 4) using Equations (5)–(7).

(5) $${Speed}_{inx}=\frac{\Delta x_{inx}}{\Delta t_{inx}}$$

where

(6) $$\Delta x={distance}_{\left(inx+DurationTime\right)}\;-\;{distance}_{inx}$$

and

(7) $$\Delta t={time}_{\left(inx+DurationTime\right)}\;-\;{time}_{inx}$$

Step 5: Gait speed segmentation

The final step involved segmentation of the walking trial into six consecutive 1 m walking segments. Gait speed was calculated separately for each segment based on the horizontal displacement of the centroid and the corresponding number of video frames required to traverse each 1 m interval (i.e., V_1_: 0–1 m, V_2_: 1–2 m, V_3_: 2–3 m, V_4_: 3–4 m, V_5_: 4–5 m, and V_6_: 5–6 m). Segment-specific gait speed was calculated from the horizontal displacement of the centroid divided by the elapsed time for each corresponding 1 m interval.

Position was sampled at the native 30 Hz frame rate, and instantaneous speed was computed over 4-frame intervals (*DurationTime* = 4), which provided a degree of temporal averaging intended to reduce sensitivity to single-frame centroid noise; no additional smoothing filter (e.g., low-pass or moving-average filter) was applied to the position-time trajectory beyond this frame-averaging step. Segment boundary-crossing frames (i.e., the 1 m interval transitions) were determined directly from the calibrated horizontal position-time trajectory.

YOLOv11x inference was performed using the Kaggle cloud-based data science platform (https://www.kaggle.com/; accessed on 27 February 2025), which provided an NVIDIA Tesla P100 GPU (16 GB memory) and an Intel^®^ Xeon^®^ CPU @ 2.30 GHz (accessed on 8 March 2025). Following body detection, gait speed estimation was implemented in MATLAB R2015a (The MathWorks, Inc., Natick, MA, USA) using the Computer Vision Toolbox and Image Processing Toolbox. Data processing was performed on a Windows 11 laptop equipped with an Intel^®^ Core™ i5-8265 U CPU @ 1.60 GHz, an NVIDIA graphics card (2 GB memory), and 24 GB DDR4 RAM (ASUSTek Computer Inc., Taipei, Taiwan).

### 2.5. Statistical Analysis

Descriptive statistics were used to summarize participant characteristics and gait speed measurements and are presented as the mean (standard deviation; SD). Data normality was assessed before inferential analyses. For each participant, gait speed values from the two trials within each walking-speed condition were averaged before statistical analysis. Therefore, Pearson correlation and Bland-Altman analyses were performed using participant-level mean values (N = 32) separately for each walking segment and walking-speed condition. Because the data were normally distributed, the concurrent validity of the proposed DL-based gait speed detection system was evaluated by calculating the Pearson’s correlation coefficient with the Tracker video analysis software (version 6.3.2). Statistical significance was set at α = 0.05 (two-sided). The strength of the correlation was interpreted as follows: r < 0.5, low; r = 0.50–0.69, moderate; r = 0.70–0.89, high; and r ≥ 0.90, very high [22]. Agreement between the proposed system and the reference method was further evaluated using Bland-Altman analysis. The mean difference (bias) and the 95% limits of agreement (LOA), calculated as the mean difference ± 1.96 SD, were used to assess the level of agreement between the two measurement methods. Narrower LOA indicated better agreement between the proposed system and the reference method [23]. All statistical analyses were performed using IBM SPSS Statistics for Windows (Version 21.0; IBM Corp., Armonk, NY, USA).

## 3. Results

### 3.1. Participant Characteristics

A total of 32 participants were enrolled in the study, comprising 12 young adults, 10 middle-aged adults, and 10 older adults. The demographic characteristics of the participants are presented in Table 1.

### 3.2. Concurrent Validity

**Figure 3:**
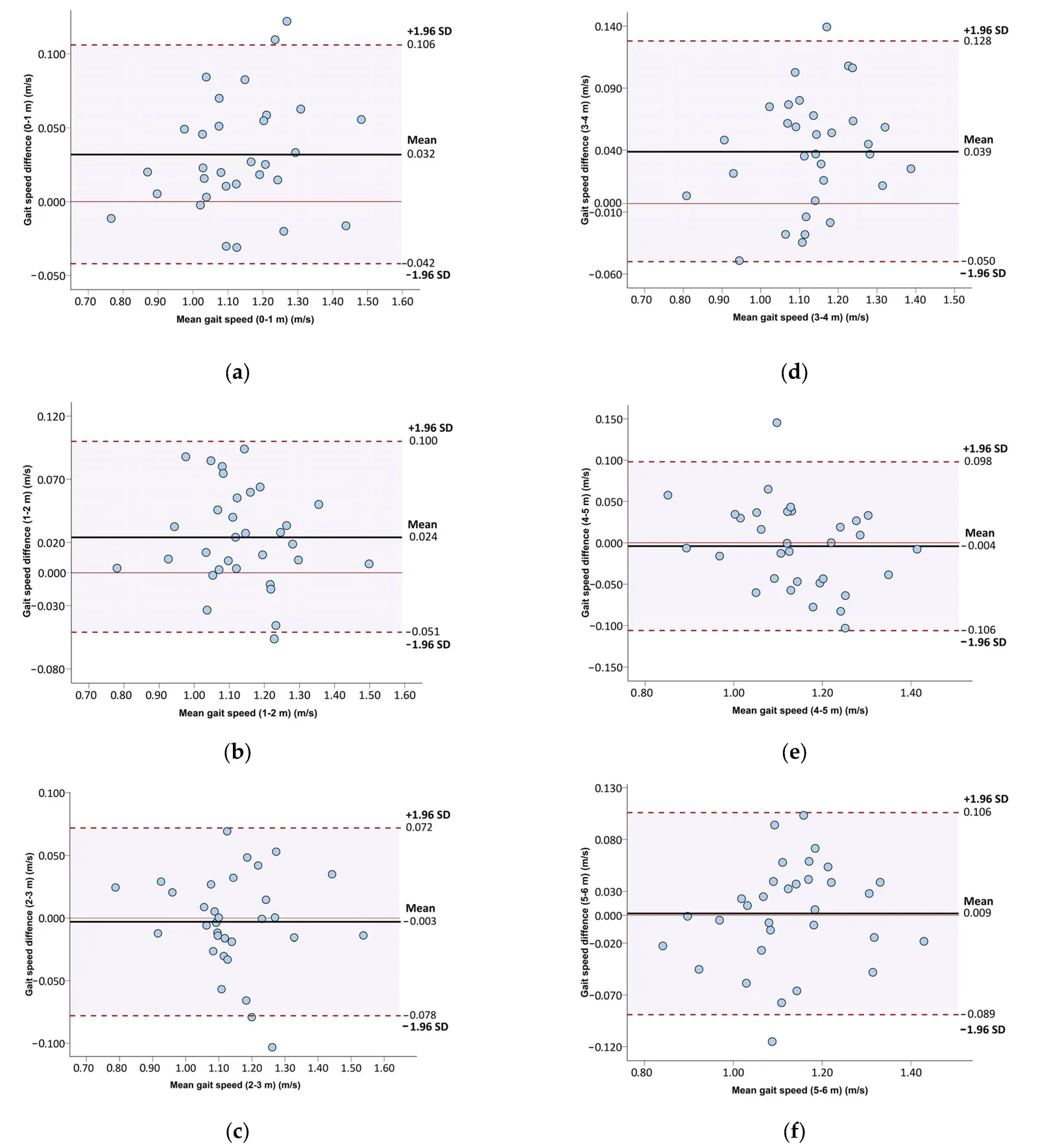
Bland-Altman plots showing the agreement between the proposed DL-based markerless video system and the Tracker video analysis software during the comfortable walking condition. The plots correspond to the six consecutive 1 m walking segments: (**a**) 0–1 m, (**b**) 1–2 m, (**c**) 2–3 m, (**d**) 3–4 m, (**e**) 4–5 m, and (**f**) 5–6 m. The x-axis represents the mean gait speed measured by the two systems, and the y-axis represents the difference in gait speed between the two systems. The solid line indicates the mean difference (bias), and the dashed lines represent the 95% LOA.

**Figure 4:**
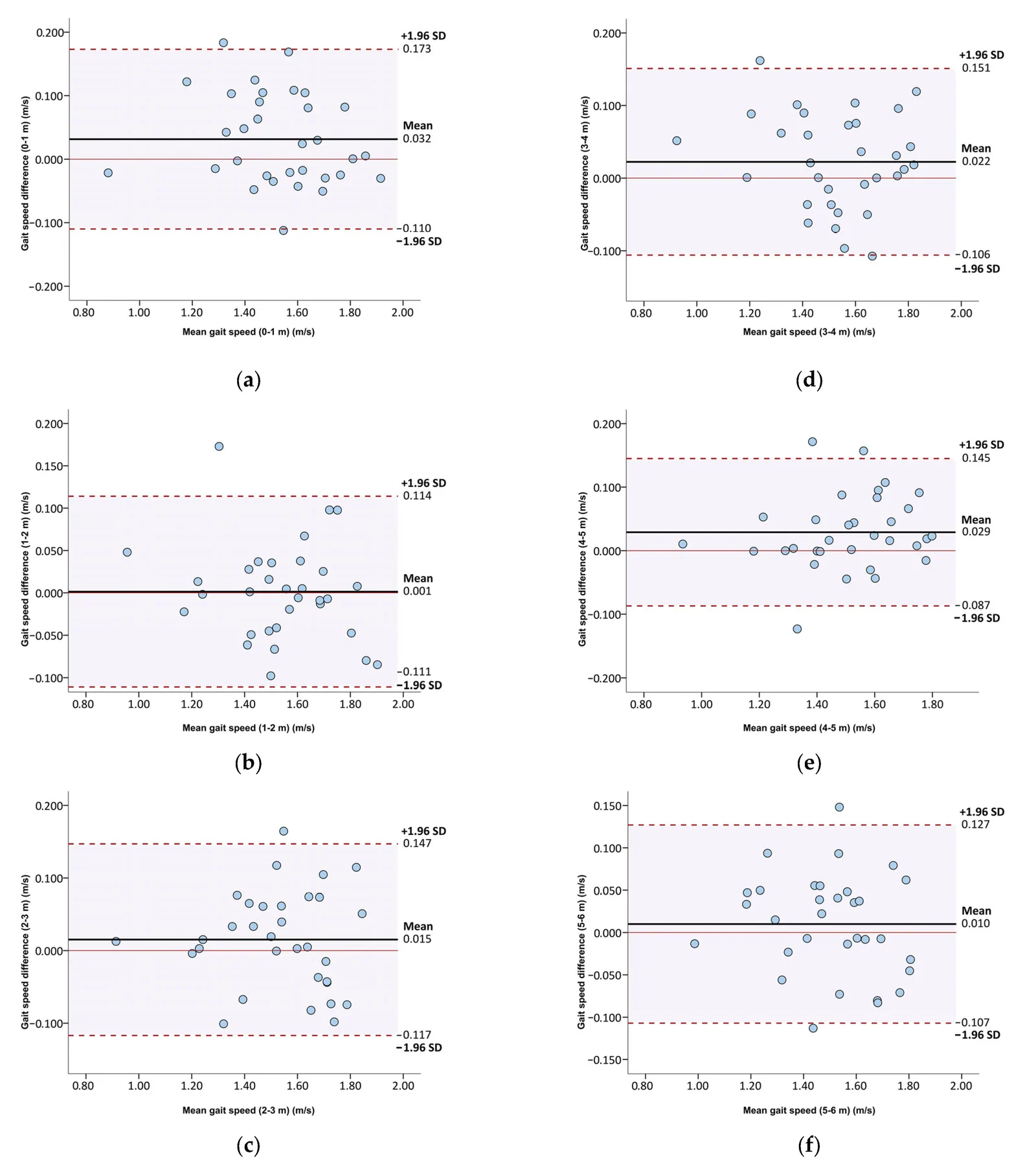
Bland-Altman plots showing the agreement between the proposed DL-based markerless video system and the Tracker video analysis software during the fast walking condition. The plots correspond to the six consecutive 1 m walking segments: (**a**) 0–1 m, (**b**) 1–2 m, (**c**) 2–3 m, (**d**) 3–4 m, (**e**) 4–5 m, and (**f**) 5–6 m. The x-axis represents the mean gait speed measured by the two systems, and the y-axis represents the difference in gait speed between the two systems. The solid line indicates the mean difference (bias), and the dashed lines represent the 95% LOA.

**Figure 5:**
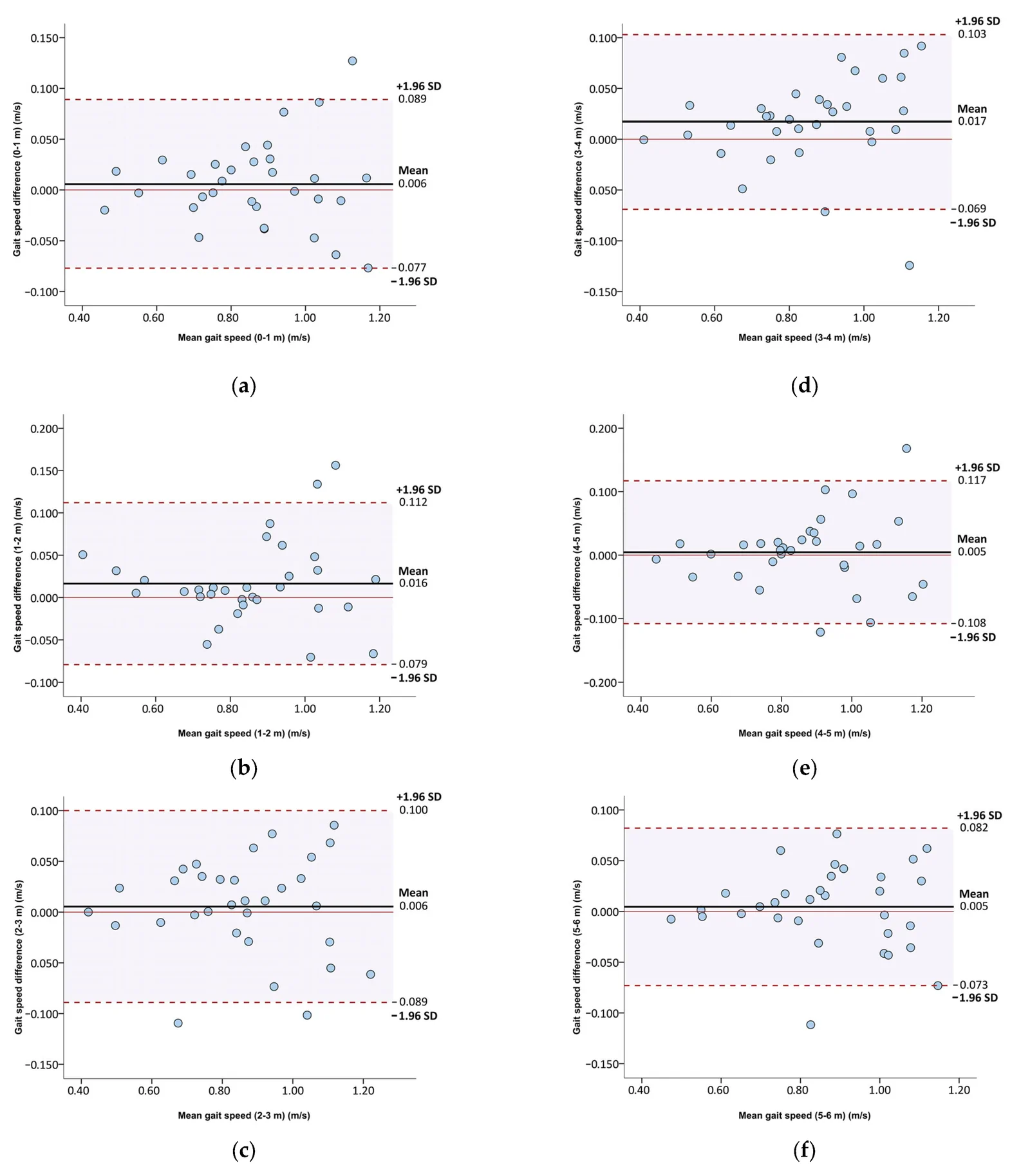
Bland-Altman plots showing the agreement between the proposed DL-based markerless video system and the Tracker video analysis software during the slow walking condition. The plots correspond to the six consecutive 1 m walking segments: (**a**) 0–1 m, (**b**) 1–2 m, (**c**) 2–3 m, (**d**) 3–4 m, (**e**) 4–5 m, and (**f**) 5–6 m. The x-axis represents the mean gait speed measured by the two systems, and the y-axis represents the difference in gait speed between the two systems. The solid line indicates the mean difference (bias), and the dashed lines represent the 95% LOA.

Pearson’s correlation coefficients between the proposed DL-based markerless video system and the Tracker video analysis software ranged from 0.92 to 0.98 across all six consecutive 1 m walking segments under comfortable, slow, and fast walking conditions, indicating very high concurrent validity (Table 2). Bland-Altman analysis demonstrated small mean differences between the two systems across all walking conditions and segments, with bias ranging from −0.004 to 0.039 m/s. The 95% LOA varied across conditions and segments, with the widest interval observed during fast walking in the 0–1 m segment (−0.110 to 0.173 m/s). The numerical Bland-Altman results are presented in Table 3, while Figure 3, Figure 4 and Figure 5 illustrate the agreement between the two systems.

## 4. Discussion

The present study evaluated the concurrent validity of a practical gait speed assessment approach that integrates DL-based body detection with spatial calibration and centroid-based tracking during the 10-MWT under outdoor non-laboratory conditions using a conventional video camera. Accordingly, the contribution of the present study lies in the practical validation of this integrated approach for gait speed estimation rather than in the development of a new deep-learning architecture or gait-analysis methodology. Overall, the proposed system demonstrated very high concurrent validity and high agreement with the reference Tracker video analysis software across all six consecutive 1 m walking segments under comfortable, slow, and fast walking conditions. These findings support our hypothesis that the proposed DL-based markerless video system can provide gait speed estimates that show high agreement with the reference method throughout the 10-MWT. Importantly, the results suggest that the system provides a practical and low-cost approach for gait speed assessment under outdoor non-laboratory conditions while maintaining high agreement with the reference method.

The high concurrent validity observed in the present study is consistent with the growing body of evidence supporting the use of markerless vision-based technologies for gait assessment. Recent systematic evidence has shown that markerless camera-based motion capture systems provide good to excellent agreement with conventional marker-based motion capture systems for most spatiotemporal gait parameters, particularly gait speed, suggesting that these technologies have matured sufficiently for quantitative gait assessment under appropriate testing conditions [13]. Likewise, Wang et al. [24] demonstrated that a single RGB camera combined with computer vision algorithms achieved strong agreement with a marker-based motion capture system for most spatiotemporal gait parameters in healthy adults, highlighting the potential of low-cost, markerless approaches as practical alternatives when conventional laboratory systems are unavailable. Our findings further extend this evidence by demonstrating that a DL-based markerless video system can estimate gait speed with high agreement with the reference video analysis method during the 10-MWT under outdoor conditions using only a conventional camera. Unlike previous validation studies that primarily evaluated markerless systems against laboratory-based motion capture systems in controlled environments, the present study compared the proposed system with Tracker video analysis software, a widely used reference method for two-dimensional motion analysis, across three walking speeds. These findings therefore broaden the current evidence supporting the clinical applicability of DL-based markerless gait assessment in an outdoor clinical setting.

The excellent agreement observed in the present study is likely attributable to the combined effects of robust DL-based object detection, accurate spatial calibration, and the relatively simple biomechanical characteristics of straight-line walking during the 10-MWT. First, the proposed system employed a DL-based object detection model (YOLOv11x), which enables robust localization of the participant across consecutive video frames. DL-based markerless motion capture has been shown to provide accurate and reliable estimation of spatiotemporal gait parameters while being more tolerant to variations in imaging conditions than conventional marker-based or feature-based approaches [13,25]. Second, gait speed was estimated from the horizontal displacement of the detected body centroid following calibration of the capture volume using reference markers. During straight-line walking, forward body translation is predominantly represented by horizontal displacement, making centroid tracking a stable and computationally efficient approach for estimating gait speed. Finally, spatial calibration allowed image coordinates to be converted into real-world distances, thereby accounting for image scale under the standardized camera setup. Collectively, these methodological characteristics likely contributed to the high level of agreement observed between the proposed system and the reference video analysis method.

An important strength of the present study is that the proposed system was validated under outdoor conditions rather than within a laboratory environment. Although laboratory-based validation is essential for establishing technical performance, the clinical implementation of gait assessment systems ultimately depends on their ability to maintain measurement performance in more practical settings. Markerless motion capture technologies have increasingly been recognized as promising tools for clinical gait assessment because they eliminate the need for wearable sensors, reflective markers, and dedicated laboratory facilities while allowing movement to be evaluated in more natural environments [13,26]. Building upon our previous work, which established the validity of a computer vision-based system in a controlled laboratory setting, the present study demonstrates that a DL-based markerless video system can maintain excellent agreement with the reference method during outdoor assessment using only a conventional camera [27]. These findings represent an important step toward translating video-based gait assessment from research laboratories into routine clinical and community practice, where simple, portable, and low-cost assessment tools are needed.

Another notable finding was that the proposed system maintained high agreement with the reference method across comfortable, slow, and fast walking conditions. This finding suggests that the system was capable of tracking changes in walking velocity over a broad range of gait speeds rather than being limited to a single self-selected walking condition. From a clinical perspective, assessing gait performance under different walking speeds provides complementary information because slow walking may reflect impaired motor control or cautious gait, whereas fast walking better reflects an individual’s locomotor capacity and physiological reserve [28,29]. The ability to quantify gait speed consistently across these conditions may therefore enhance the clinical utility of the proposed system for comprehensive mobility assessment. Furthermore, the present study estimated gait speed within six consecutive 1 m walking segments, allowing temporal changes in walking velocity to be examined throughout the 10-MWT. This segment-based approach may provide more detailed information than a single average gait speed by characterizing segment-to-segment variations in walking velocity throughout the central 6 m section, thereby offering additional insights into walking performance that may not be apparent from an overall gait speed alone. However, the present study did not determine whether these segment-to-segment variations provide clinically meaningful information beyond conventional average gait speed. Therefore, segment-specific gait speed should currently be considered an exploratory feature of the proposed system that warrants further investigation.

The present study has strengths that support the clinical relevance of the proposed system. Unlike many previous validation studies conducted exclusively under controlled laboratory conditions, the proposed DL-based markerless video system was evaluated under outdoor non-laboratory conditions using only a conventional RGB video camera. In addition, the system demonstrated consistently high agreement with the reference method across comfortable, slow, and fast walking conditions while providing gait speed estimates across six consecutive 1 m walking segments, thereby allowing temporal changes in walking velocity throughout the 10-MWT to be characterized. These features highlight the potential of the proposed approach as a simple, low-cost, and accessible tool for quantitative gait assessment in clinical and community settings. Nevertheless, several limitations should be acknowledged. The proposed system was validated against two-dimensional Tracker video analysis software rather than an independent biomechanical reference system. Therefore, the present findings demonstrate agreement with the reference video analysis method under the tested conditions and should not be interpreted as establishing absolute measurement accuracy. Future studies should validate the proposed system against an independent reference system, such as an instrumented walkway or laboratory-based three-dimensional motion capture system. In addition, an a priori clinically acceptable error threshold was not prespecified; therefore, the clinical acceptability of the observed measurement differences cannot be established from the present study and should be evaluated in future studies involving specific clinical populations. The pixel-to-distance conversion was also based on a two-point linear calibration along the horizontal walking axis rather than a full camera calibration incorporating lens distortion correction or homography-based perspective correction; although the fixed, perpendicular camera geometry and standardized walkway were intended to minimize such effects, small deviations in walking path or participant depth relative to the calibrated plane could introduce measurement error that was not directly quantified in this study. Similarly, the use of the bounding-box centroid, rather than the anatomical center of mass, as the position reference is a further methodological simplification, since centroid position may be influenced by arm swing, trunk sway, clothing, or partial occlusion. Furthermore, agreement between the proposed system and the reference method was not uniform across the six 1 m segments; the widest limits of agreement were observed in the initial 0–1 m segment during fast walking, indicating that segment-level estimates, particularly near the start of the measured zone, should be interpreted with more caution than the overall 6 m average speed, and should currently be regarded as an exploratory feature of the proposed system. The study also included only healthy participants, which limits the generalizability of the findings to individuals with pathological gait patterns. Furthermore, gait assessment was limited to straight-line walking under daylight outdoor conditions, and only gait speed was evaluated. Future studies should therefore further evaluate the validity of the proposed system across a wider range of outdoor non-laboratory environments with varying background complexity. Validation should also be extended to clinical populations, including individuals with pathological gait patterns or those who use walking aids, to establish the robustness and generalizability of the system. In addition, future development should focus on integrating the proposed system into user-friendly digital platforms, such as mobile or web-based applications, to facilitate its implementation in routine clinical practice, community health services, and home-based monitoring. Despite these limitations, the findings demonstrate that the proposed DL-based markerless video system provides a practical and low-cost approach for gait speed assessment during the 10-MWT, with high agreement with the reference video analysis method. With further validation in clinical populations and more diverse testing environments, this approach may have potential for broader clinical and community-based applications [30].

## 5. Conclusions

The proposed DL-based markerless video system demonstrated high concurrent validity and agreement with Tracker video analysis software for gait speed estimation during the 10-MWT in healthy adults under standardized outdoor non-laboratory conditions. Further validation against independent biomechanical reference systems and in clinical populations is required before its application in routine clinical practice can be established.

## Figures and Tables

**Table 1:** Demographic characteristics of participants by age group.

Characteristic	Young Adults (n = 12)	Middle-Aged Adults (n = 10)	Older Adults (n = 10)
Gender (n)			
Male	2	6	4
Female	10	4	6
Age (yrs)	29.67 (6.91)	50.50 (3.95)	69.80 (5.51)
Height (m)	1.62 (0.09)	1.67 (0.11)	1.64 (0.07)
Weight (kg)	67.50 (21.30)	76.30 (14.75)	62.65 (15.72)
BMI (kg/m^−2^)	25.68 (8.05)	27.36 (4.85)	23.13 (4.04)

Note: Data are presented as mean (SD) unless otherwise indicated. BMI = body mass index.

**Table 2:** Gait speed measurements and Pearson’s correlation coefficients between the proposed DL-based markerless video system and the Tracker video analysis software during the 10-MWT.

Walking Segment (m)	Walking Speed Condition (N = 32)								
	Comfortable Walking (m/s)			Slow Walking (m/s)			Fast Walking (m/s)		
	Tracker	Deep Learning	r	Tracker	Deep Learning	r	Tracker	Deep Learning	r
	Mean (SD)	Mean (SD)		Mean (SD)	Mean (SD)		Mean (SD)	Mean (SD)	
0–1	1.15 (0.16)	1.12 (0.15)	0.97	0.87 (0.19)	0.86 (0.19)	0.98	1.54 (0.21)	1.51 (0.22)	0.95
1–2	1.15 (0.14)	1.12 (0.14)	0.96	0.86 (0.20)	0.85 (0.19)	0.97	1.54 (0.21)	1.54 (0.22)	0.97
2–3	1.14 (0.14)	1.14 (0.15)	0.97	0.86 (0.20)	0.85 (0.20)	0.97	1.54 (0.21)	1.53 (0.21)	0.95
3–4	1.15 (0.13)	1.11 (0.12)	0.94	0.87 (0.20)	0.85 (0.19)	0.98	1.54 (0.21)	1.52 (0.22)	0.95
4–5	1.14 (0.12)	1.14 (0.14)	0.92	0.87 (0.20)	0.87 (0.19)	0.96	1.52 (0.20)	1.50 (0.19)	0.96
5–6	1.13 (0.14)	1.12 (0.13)	0.93	0.87 (0.18)	0.87 (0.18)	0.98	1.51 (0.20)	1.50 (0.21)	0.96

Note: Tracker = reference video analysis software; Deep learning = proposed DL-based markerless video system; r = Pearson’s correlation coefficient. All correlations were statistically significant (*p* < 0.001).

**Table 3:** Bland-Altman agreement between the proposed DL-based markerless video system and Tracker video analysis software across walking-speed conditions and 1 m walking segments.

Walking Segment (m)	Comfortable Bias	95% LOA	Slow Bias	95% LOA	Fast Bias	95% LOA
0–1	0.032	−0.042 to 0.106	0.006	−0.077 to 0.089	0.032	−0.110 to 0.173
1–2	0.024	−0.051 to 0.100	0.016	−0.079 to 0.112	0.001	−0.112 to 0.114
2–3	−0.003	−0.078 to 0.072	0.006	−0.089 to 0.100	0.015	−0.117 to 0.147
3–4	0.039	−0.050 to 0.128	0.017	−0.069 to 0.103	0.022	−0.106 to 0.151
4–5	−0.004	−0.106 to 0.098	0.005	−0.108 to 0.117	0.029	−0.087 to 0.145
5–6	0.009	−0.089 to 0.106	0.005	−0.073 to 0.082	0.010	−0.107 to 0.127

Note: Bias was calculated as Tracker—DL-based markerless video system. LOA = limits of agreement. Values are expressed in m/s.

## Data Availability

The original contributions presented in this study are included in the article. Further inquiries can be directed to the corresponding author.

## Abbreviations

The following abbreviations are used in this manuscript:

10-MWT	10-meter walk test
BMI	Body mass index
COCO	Common Objects in Context
DL	Deep learning
HD	High definition
LOA	Limits of agreement
RGB	Red, green, blue
SD	Standard deviation
YOLO	You Only Look Once
